# The importance of including ADHD in the differential diagnosis in adults. About a case

**DOI:** 10.1192/j.eurpsy.2024.1707

**Published:** 2024-08-27

**Authors:** E. González Laynez, B. Gamo Bravo, S. M. Bañón González, N. Ogando Portilla

**Affiliations:** ^1^Psychiatry, Hospital Universitario de Toledo, Toledo; ^2^Psychiatry, Hospital Universitario Infanta Sofía, Madrid, Spain

## Abstract

**Introduction:**

ADHD is a diagnosis almost always made in childhood or adolescence and oftent difficult to make it new in adults because it is not thought of in the differential diagnosis process and for the lack of experience from adult devices.

- ADHD in adults is characterized by symptoms of executive dysfunction, inattention, emotional dysregulation. The symptoms of impulsivity and hyperactivity tend to be less evident.

**Objectives:**

- Frequently, the adult patient with ADHD comes to the consultation with a secondary symptom and the primary pathology is hidden and often not evident at first glance.

**Methods:**

- A 20-year-old woman, university student, with no relevant medical or psychiatric history, without toxic habits, who attended her first consultation referred by her primary care physician for long-standing insomnia, restless legs and anxiety.
- The patient’s underlying complaint and her bigger concern is her poor academic performance. It is striking that she has just started her third year at the university, the first year she did not pass any subjects, the second year she changed majors and only passed two, now she is repeating the course.
- The examination did not reveal overt affective symptoms, nor psychotic symptoms or other notable psychopathology. The patient’s speech tended towards superficiality, inconcretion, it was salty, it was difficult for her to express herself, even suggesting a certain intellectual disability.
- In the first consultation sleep study is requested. And referral to clinical psychology consultation for psychometric study.

**Results:**

- She is administered Clinical interview and WAIS IV (Adult Intelligence Scale), Trail Making Test, d2 and Stroop, Diagnostic Interview for ADHD in adults (inattention items).
- The WAIS-IV demonstrated global cognitive abilities within normality, although with significantly lower scores in the IMT and IVP indices which involve the functions of attention, concentration, mental control and short-term visual memory). Trail Making Test, d2, Stroop and Diagnostic Interview of ADHD in adults (inattention items) yielded profile results highly suggestive of Attention Deficit Disorder without Hyperactivity.
- The insomnia subsided with a regimen of 7.5 mg of mirtazapine per day. Subsequently, after starting treatment with low-dose methylphenidate (20 mg/day), the patient improved very significantly, both academic performance and social functioning and mood, self-esteem and subjective well-being.

**Conclusions:**

- We must always include ADHD in the differential diagnosis of a young adult patient when faced with a wide variety of consultation symptoms, especially if they report some type of deterioration or dysfunction in their social, family or academic life.
- An adequate clinical evaluation supported, if possible, by psychometric tests is essential to reach the diagnosis, which allows establishing an effective treatment that modifies the patient’s overall prognosis.

**Disclosure of Interest:**

None Declared

